# Associations of Genetic Variants at Nongenic Susceptibility Loci with Breast Cancer Risk and Heterogeneity by Tumor Subtype in Southern Han Chinese Women

**DOI:** 10.1155/2016/3065493

**Published:** 2016-02-28

**Authors:** Huiying Liang, Hong Li, Xuexi Yang, Lujia Chen, Anna Zhu, Minying Sun, Changsheng Ye, Ming Li

**Affiliations:** ^1^Institute of Pediatrics, Guangzhou Women and Children's Medical Center, Guangzhou Medical University, Jinsui Road 9, Tianhe District, Guangzhou, Guangdong 510623, China; ^2^School of Biotechnology, Southern Medical University, Shatai Southern Road 1023, Baiyun District, Guangzhou, Guangdong 510515, China; ^3^Breast Center Nanfang Hospital, Southern Medical University, Shatai Southern Road 1023, Baiyun District, Guangzhou, Guangdong 510515, China; ^4^Department of Primary Public Health, Guangzhou Center for Disease Control and Prevention, Qide Road 1, Baiyun District, Guangzhou, Guangdong 510440, China

## Abstract

Current understanding of cancer genomes is mainly “gene centric.” However, GWAS have identified some nongenic breast cancer susceptibility loci. Validation studies showed inconsistent results among different populations. To further explore this inconsistency and to investigate associations by intrinsic subtype (Luminal-A, Luminal-B, ER−&PR−&HER2+, and triple negative) among Southern Han Chinese women, we genotyped five nongenic polymorphisms (2q35: rs13387042, 5p12: rs981782 and rs4415084, and 8q24: rs1562430 and rs13281615) using MassARRAY IPLEX platform in 609 patients and 882 controls. Significant associations with breast cancer were observed for rs13387042 and rs4415084 with OR (95% CI) per-allele 1.29 (1.00–1.66) and 0.83 (0.71–0.97), respectively. In subtype specific analysis, rs13387042 (per-allele adjusted OR = 1.36, 95% CI = 1.00–1.87) and rs4415084 (per-allele adjusted OR = 0.82, 95% CI = 0.66–1.00) showed slightly significant association with Luminal-A subtype; however, only rs13387042 was associated with ER−&PR−&HER2+ tumors (per-allele adjusted OR = 1.55, 95% CI = 1.00–2.40), and none of them were linked to Luminal-B and triple negative subtype. Collectively, nongenic SNPs were heterogeneous according to the intrinsic subtype. Further studies with larger datasets along with intrinsic subtype categorization should explore and confirm the role of these variants in increasing breast cancer risk.

## 1. Introduction

Detection and characterization of the genetic diversity of disease-associated loci are a major emphasis of current scientific inquiry in cancer. Rapid technological advances have enabled us to explore the increasingly complex genetic architectures and their relationship to cancer. However, current understanding of cancer genomes is primarily a “gene centric” view [1]. Because compared to genetic variants located outside genes, genic variants are frequently judged to be more likely to alter gene function and affect disease risk [2].

Focusing only on genic variants is obviously not a comprehensive strategy for research into the genetics of cancer, due to the fact that the majority lie in nongenic regions [3]. Consistent with this theory, several recent large-scale genome-wide association studies (GWAS) of breast cancer [4–6] have identified three novel genetic susceptibility loci (8q24, 2q35, 5p12) that are associated with the risk of breast cancer. None of these loci were in coding regions of genes, and three variants (8q24: rs13281615, 2q35: rs13387042, and 5p12: rs10941679), each of which reflected a genetically independent locus, show independent associations with risk of breast cancer, although statistical gene-gene interactions resulting in larger joint effects than expected by their individual relative risks could exist.

Associations between three nongenic loci SNPs and breast cancer risk have been independently replicated by subsequent studies in recent years among East Asians, Africans, and some other ethnic populations; however, a proportion of them have yielded conflicting or inconclusive results [7–9]. The reasons for the differences in results remain to be determined. Growing evidence suggests substantial heterogeneity for association with the polymorphism of hormone-receptor defined subtypes of breast cancer [10].

In addition, all four GWAS were conducted in European ancestry (EA) populations, who differ from women of other ethnic groups in certain aspects of their genetic architecture. In this regard, it is important to validate these findings in other ethnic populations and perhaps use the different linkage disequilibrium (LD) patterns observed in non-European ancestral population to refine associated genomic regions. In our previous studies [11–13], most genic single nucleotide polymorphisms (SNPs)/loci identified from previous GWAS in populations of European descent had been evaluated in Southern Han Chinese women with an urbanized lifestyle, with breast cancer rates approaching those of the West [14]. Distinctively, the ethnic Chinese in Southern China (mainly Teochew and Cantonese) represent a geographically distinct population. To date, no report has associated above-mentioned nongenic loci with breast cancer risk in Southern Han Chinese women.

Herein, we evaluated the association of three nongenic loci with breast cancer risk in Southern Han Chinese women. Furthermore, the associations of these loci with four breast cancer subtypes defined by four markers (estrogen receptor (ER) status, progestin receptor (PR) status, human epidermal growth factor receptor-2 (HER-2) status, and Ki-67 expression status) were also evaluated. Without a doubt, this paper will expand and refine our previous reports on analyses mainly focusing on genic loci and thus make more definite conclusions than previous reports.

## 2. Materials and Methods

### 2.1. Study Population

Individuals included in the current analysis were Han Chinese women who participated in the Southern China Breast Cancer Genetics Study (SCBCGS) [15]. The SCBCGS was a multicenter, hospital-based study of breast cancer conducted among Han Chinese women from three areas of the Southern China, including Canton, Chongqing, and Nanchang. Database review of the SCBCGS identified 609 breast cancer patients with detailed and complete information on ER, PR, HER2, and Ki-67 expression status and 882 ethnicity, age (±5 years), and community of residence matched controls for present study. Detailed information on histories of menstrual and reproductive factors, hormone therapy (HT), weight, height, and family history of breast cancer for each participant was collected during in-person interviews conducted as part of the SCBCGS. After written informed consent was obtained, a peripheral blood sample was collected from each participant. The study was approved by the Ethical Committee of Southern Medical University and was conducted in accordance with the Declaration of Helsinki.

Our previous study has shown the specific characteristics of the controls and cases by the intrinsic subtype [15]. Briefly, compared with controls, cancer cases were older and more likely to be parous with first full-term pregnancy at ≥30 years and postmenopausal HT nonuser. Notably, no significant differences were seen in basic characteristics between subtypes.

### 2.2. SNP Selection and Genotyping

From 2009 to 2013, we collaborated with other two research groups to start validating breast cancer susceptibility genes (including nongenic loci) in Chinese cohorts. As far, recent GWAS have identified more than 20 different intergenic loci associated with breast cancer risk, which can be viewed in the NHGRI GWAS Catalog. However, seven GWAS [4, 5, 16–20] published before 2013 just identified a total of 8 SNPs in 6 nongenic loci associated with breast cancer in almost European decent populations. Three SNPs from the GWAS of Murabito et al. [18] were excluded because of missing replication in another independent cohort for Stage 2 of GWAS and unknown risk alleles. Thus, only 5 SNPs showing statistically significant associations with breast cancer were selected for analysis in this study. These 5 SNPs represent 3 independent loci that are present in intergenic regions, specifically, one SNP at 2q35 (rs13387042), two SNPs at 5p12 (rs981782 and rs4415084), and two SNPs at 8q24 (rs1562430 and rs13281615). These index SNPs had reached significance in one or more replication and refinement studies of European, Asian, and/or African ancestry populations [7–9]. The genotyping of SNPs was done using the SEQUENOM MassARRAY matrix-assisted laser desorption ionization time of flight mass spectrometry platform [12, 21].

### 2.3. Classification of Biologic Subtype

Four subtypes were constructed based on the receptor status of the primary tumor, specifically, (i) triple negative (ER−, PR−, HER2−), (ii) ER−&PR−&HER2+ (ER−, PR−, HER2+), (iii) Luminal-B (ER+ and/or PR+ and either HER2+ and/or Ki67^high^), and (iv) Luminal-A (ER+ and/or PR+ and not HER2+ or Ki67^high^). The number of cases with above-mentioned tumor marker data available, the classification scheme we used based on combinations of the marker, and specific characteristics of the controls and cases classified according to the intrinsic subtype have been described in our previous reports [15].

### 2.4. Statistical Analysis

For each SNP, deviation of genotype frequencies in controls from the Hardy Weinberg Equilibrium (HWE) was assessed by a goodness-of-fit *χ* ^2^ test. Differences in frequencies of SNP alleles and genotypes between cases and controls were evaluated using chi-square test or Fisher's exact test as appropriate. Breast cancer risk was estimated as odds ratios (ORs) and 95% confidence intervals (95% CIs), based on unconditional logistic regression and adjusted for potential confounders including age, age at first full-term pregnancy, menopausal status, and hormonal therapy use [15]. Analyses were carried out assuming a dominant, codominant, and additive allelic effect for each polymorphism.

Stratified analysis according to the 4 breast cancer subtypes was additionally conducted. To correct multiple testing, we estimated the adjusted significance by applying the Bonferroni correction for all the SNPs tested in the analysis. All statistical tests were two-sided, and *P* < 0.05 was considered statistically significant. Statistical analysis was performed using SPSS version 19.0 (IBM SPSS Statistics for Windows, IBM Corporation, Somers, NY) unless otherwise specified.

## 3. Results

### 3.1. Hardy Weinberg Equilibrium Testing

We genotyped five nongenic SNPs in 609 breast cancer cases and 882 controls. The minor allele frequencies of all tested SNPs in Southern Han Chinese are roughly similar with the corresponding frequencies of the HapMap HCB (Chinese) and JPT (Japanese) population. All the observed genotype frequencies were found to be in agreement with HWE in the controls except for rs13281615, which deviates from HWE (*P* < 1 × 10^−4^) and thus was excluded from the subsequent analyses (Table 1).

### 3.2. Association between SNPs and Breast Cancer Risk

Table 2 shows the allele and genotype distributions of non-genic rs13387042, rs981782, rs4415084, and rs1562430 polymorphisms in the combined sample. Univariate analysis showed that rs13387042 (per-allele OR = 1.34, 95% CI = 1.05–1.72) and rs4415084 (per-allele OR = 0.83, 95% CI = 0.72–0.97) were significantly correlated with the risk of breast cancer. After adjusting for age, age at first full-term pregnancy, menopausal status, hormonal therapy use, and logistic regression analysis further confirmed these associations which remained significant in per-allele model for rs13387042 (OR = 1.29, 95% CI = 1.00–1.66) and rs4415084 (OR = 0.83, 95% CI = 0.71–0.97).

### 3.3. Associations with Breast Cancer Risk by Subtype Separately

In subgroup of Luminal-A cases, a significant OR of 1.43 (95% CI = 1.05–1.95, *P* = 0.03) was found for rs13387042-A variant. Significant results were also observed using recessive (OR = 5.01, 95% CI = 1.40–17.91, *P* = 0.01) and codominant genetic model (homozygous: OR = 5.23, 95% CI = 1.46–18.70, *P* = 0.01). After adjusting for potential cofounders including age, age at first full-term pregnancy, menopausal status, and hormonal therapy use, these findings persisted (per-allele adjusted OR = 1.36, 95% CI = 1.00–1.87) (Table 3). Furthermore, rs4415084 also showed marginally significant association with Luminal-A breast cancer after adjusting for potential cofounders (per-allele adjusted OR = 0.82, 95% CI = 0.66–1.00).

For ER−&PR−&HER2+ cases, the heterozygous genotype (G/A) of rs13387042 significantly increased breast cancer risk under the codominant (adjusted OR = 1.68, 95% CI = 1.07–2.65, *P* = 0.03), dominant (adjusted OR = 1.64, 95% CI = 1.04–2.58, *P* = 0.04), and overdominant models (adjusted OR = 1.69, 95% CI = 1.07–2.67, *P* = 0.03) with an A-allelic trend of OR = 1.55 (95% CI = 1.00–2.40) after adjusting for potential cofounders (Table 4).

For Luminal-B and triple negative breast cancer cases, however, no significant associations were found with the four nongenic variants under all five genetic models (*P* > 0.05) (see Supplementary Table S1 and Supplementary Table S2 in Supplementary Material available online at http://dx.doi.org/10.1155/2016/3065493).

## 4. Discussion

Understanding the genetic basis of disease can transform medicine by elucidating relevant biochemical pathways for drug targets and by enabling personalized risk assessments, but medical research has focused primarily on genic variants, owing to the difficulty of interpreting nongenic mutations. However, a survey of human trait-associated SNPs found that most are located in noncoding regions (43% from nongenic regions and 45% from introns), suggesting that the search for functional polymorphisms should extend beyond genic regions [22].

Furthermore, studies trying to investigate the association between common nongenic polymorphisms with breast cancer susceptibility have yielded inconsistent results [23]. There are some points that should be concerned for such inconsistent results. Firstly, ethnic differences may attribute to these different results, since the distributions of the studying polymorphism were different between various ethnic populations. For instance, the MAF differs from Chinese population, Whites, to African descents (Table 1). On the other hand, a polymorphism may be in close linkage with another nearby causal variant in one ethnic population but not in another. Furthermore, study design or small sample size or some environmental factors may also affect the results. Regretfully all these studies did not consider intrinsic subtypes of the breast cancers in study design. It is possible that a positive SNP association with breast cancer in individuals mainly composed of one specific subtype that may be negative in another study population mainly consisting of another subtype [24].

Thus, present study investigated whether 5 common nongenic SNPs located in “gene desert” regions were associated with specific tumor subtypes defined by four markers (ER, PR, HER2, and Ki67). This will be the first Chinese study to validate and provide convincing evidence for heterogeneity in the strength of the associations of nongenic susceptibility loci with respect to the risk of tumor subtypes. Furthermore, stratification of tumors also provided further insights into etiological heterogeneity.

First, this study confirmed that two nongenic SNPs (rs13387042 and rs4415084) were significantly associated with increased risk of breast cancer. Rs13387042 was first identified as a breast cancer susceptibility SNP in two GWAS conducted among Europeans [5, 19]. Significant associations were subsequently confirmed in the later studies on Europeans and African American women [25, 26]. However, the findings were inconsistent in Chinese women. For example, Dai et al. reported significant association with increased breast cancer risk [27], whereas Zheng et al. did not find significance [28].

For rs4415084, the association with breast cancer has been evaluated by three studies in Chinese since it was first identified through GWAS approach [6]; however, all three studies yielded nonsignificant results [27, 29, 30]. The findings from a systematic review [23] including three above-mentioned studies on Chinese women, however, are consistent with our present results.

Further subtype stratification analyses showed that rs13387042 and rs4415084 marginally associated with Luminal-A breast cancer even after adjusting for potential cofounders including age, age at first full-term pregnancy, menopausal status, and hormonal therapy use (Table 3). However, only rs13387042 was statically significant with HRE2 overexpression breast cancer cases (Table 4). Beyond the significant associations mentioned above, no significant associations were detected between all four nongenic SNPs and the Luminal-B (Table S1) and triple negative (Table S2) breast cancer subtypes in Southern Han Chinese population.

Finding the potential biological functions of such SNPs can be an important step towards further study. However, we identified that 2q35-rs13387042 and 5p12-rs4415084 are located in a 90-kb and a 100 kb LD block containing neither known genes nor noncoding RNAs, respectively. Furthermore, both SNPs are located more than 100 kb from the nearest gene: TNP1 and MRPS30, respectively. The causal variant in this region has not been determined. Thus, functional studies in these regions are likely to lead to a better understanding of mechanisms of carcinogenesis and progression of breast cancer. In addition, the ORs we obtained were small with narrow CIs indicating that when considered alone as a genetic factor, both polymorphisms have a very small effect on susceptibility to breast cancer.

A strength of our study was that ER, PR, HER-2, and ki-67 status were all assessed using the same processing protocols and criteria for pathology review for all cases. However, three important limitations of this study must be considered. First, we could not confirm that other SNPs lacked an association with specific breast cancer subtypes because we had limited samples and a lack of power to detect a true association. For example, though current study has sufficient power (>90%) to detect a log-additive OR of 1.30 with allele frequencies >27%, the exact powers to detect a log-additive OR of 1.30 of four selected SNPs (rs13387042, rs981782, rs4415084, and rs1562430) with Luminal-B subtypes using the MAF in Table 1 were 20.6%, 44.5%, 45.8%, and 32.2%, respectively. Furthermore, considering the sample size and the marginal effect size, multiple comparison was also performed. However, based on the multiple hypothesis testing, all these associations were not significant (all Bonferroni-adjusted *P* > 0.05). Thus, larger sample sizes could help improve the power and ensure the correct conclusion regarding whether these SNPs are associated with specific breast cancer subtypes.

Additionally, we cannot rule out that other nongenic loci may be risk factors for breast cancer and specific subtypes because there may be different functional variants among different populations and specific subtypes. For example, one GWAS among Chinese women by Zheng et al. in 2010 identified a novel SNP rs2046210 at a nongenic loci of 6q25.1 exhibiting strong and consistent significant association with breast cancer across all three stages in Chinese women only [28]. Difference in the LD patterns between the significant and hidden functional variants may be another unignored possibility. For example, in the HapMap CEU population, the rs4415084 SNP resides in a LD block that is nearly 100 kb covering some gaps of low LD. In the HapMap CHB population, this big LD block was split into smaller blocks (66 kb). Thus, genotyping other known SNPs in these nongenic regions according to HapMap data may help elucidate the basis for potential ethnicity/subtype-related disparities and provide a better understanding of the genetic basis of specific breast cancer subtypes.

Third, misclassification of breast cancer subtypes is likely to be independent of susceptibly loci and thus would tend to underestimate association strengths rather than create spurious associations [29]. For example, a recent study showed a high discordance between HER2 expression based on IHC and mRNA; 60% of the tumors classified as HER2+ by IHC did not display elevated levels by mRNA expression [30]. Finally, only subtype specific analysis was conducted in present study, but there may be other interactions between gene and environment factors. Thus, further studies about gene-environment interaction on breast cancer could be useful.

## 5. Conclusions

Collectively, our study demonstrated a marginally significant association of nongenic SNPs with breast cancer risk in Southern Chinese Han population and added evidences for differential susceptibility according to intrinsic subtype. Moreover, further investigation of larger data sets along with intrinsic subtype categorization and functional studies are required to determine how and to what degree these variant gene forms are influencing breast cancer pathogenesis.

## Supplementary Material

Supplementary materials included two tables showing the results from both the univariate and multivariate logistic regression analyses on the association between four nongenic polymorphisms and Luminal-B/triple negative subtypes.

## Figures and Tables

**Table 1:** Minor allele frequency distribution in different ethnic groups from HapMap and Southern Han Chinese women.

SNP	Position^*∗*^	Allele (major/minor)	Minor allele frequency distribution					HWEP
			Present	HCB	JPT	CEU	YRI	
rs13387042	chr2: 217041109	G/A	9.0%	11.6%	11.6%	56.2%	76.1%	0.30
rs981782	chr5: 45285616	T/G	36.7%	32.6%	40.7%	38.7%	0.0%	0.25
rs4415084	chr5: 44662413	T/C	45.1%	47.7%	45.3%	62.4%	36.2%	0.06
rs1562430	chr8: rs1562430	A/G	17.9%	16.3%	15.1%	35.3%	49.1%	0.57
**rs13281615**	**chr8: 127343372**	**G/A**	**40.0%**	**44.4%**	**38.9%**	**54.2%**	**56.7%**	**<0.0001 **

HCB, Han Chinese in Beijing; JPT, Japanese in Tokyo; CEU, European descent from Utah; YRI, Yoruba individuals from Ibadan, Nigeria; HWEP, Hardy Weinberg Equilibrium *P* value. ^*∗*^According to the new human reference assembly (GRCh38.p2).

**Table 2:** Association between four nongenic polymorphisms and breast cancer risk.

SNP or model	Genotype or allele	Controls (%) (*n* = 882)	Cases (%) (*n* = 609)	Raw OR (95% CI)	Raw *P*	Adjusted OR (95% CI)^*∗*^	Adjusted *P*
rs13387042							

Codominant model	G/G	704 (82.5%)	472 (77.9%)	Reference		Reference	
	G/A	145 (17.0%)	128 (21.1%)	1.32 (1.01–1.72)	0.04	1.27 (0.97–1.67)	0.08
	A/A	4 (0.5%)	6 (1.0%)	2.24 (0.63–7.97)	0.21	1.96 (0.54–7.13)	0.31
Dominant model	G/A + A/A	149 (17.5%)	134 (22.1%)	1.34 (1.03–1.74)	0.03	1.29 (0.99–1.69)	0.06
Recessive model	G/G + G/A	849 (99.5%)	600 (99.0%)	Reference		Reference	
	A/A	4 (0.5%)	6 (1.0%)	2.12 (0.60–7.55)	0.24	1.87 (0.52–6.80)	0.33
Overdominant model	G/G + A/A	708 (83.0%)	478 (78.9%)	Reference		Reference	
	G/A	145 (17.0%)	128 (21.1%)	1.31 (1.00–1.70)	0.048	1.27 (0.97–1.66)	0.08
Log-additive model	G	1553 (91.0%)	1072 (88.4%)	Reference		Reference	
	A	153 (9.0%)	140 (11.6%)	1.34 (1.05–1.72)	0.02	1.29 (1.00–1.66)	0.046

rs981782							

Codominant model	T/T	360 (41.0%)	252 (41.4%)	Reference		Reference	
	G/T	392 (44.6%)	260 (42.7%)	0.95 (0.76–1.19)	0.64	0.96 (0.76–1.20)	0.71
	G/G	127 (14.4%)	97 (15.9%)	1.09 (0.80–1.49)	0.58	1.08 (0.79–1.48)	0.61
Dominant model	G/T + G/G	519 (59.0%)	357 (58.6%)	0.98 (0.80–1.21)	0.87	0.99 (0.80–1.22)	0.92
Recessive model	T/T + G/T	752 (85.5%)	512 (84.1%)	Reference		Reference	
	G/G	127 (14.4%)	97 (15.9%)	1.12 (0.84–1.49)	0.43	1.11 (0.83–1.48)	0.49
Overdominant model	T/T + G/G	487 (55.4%)	349 (57.3%)	Reference		Reference	
	G/T	392 (44.6%)	260 (42.7%)	0.93 (0.75–1.14)	0.47	0.94 (0.76–1.16)	0.55
Log-additive model	T	1112 (63.3%)	764 (62.7%)	Reference		Reference	
	G	646 (36.7%)	454 (37.3%)	1.02 (0.88–1.18)	0.78	1.02 (0.88–1.19)	0.78

rs4415084							

Codominant model	T/T	244 (28.4%)	208 (34.3%)	Reference		Reference	
	C/T	453 (52.8%)	300 (49.5%)	0.78 (0.61–0.98)	0.04	0.77 (0.60–0.98)	0.03
	C/C	161 (18.8%)	98 (16.2%)	0.71 (0.52–0.98)	0.03	0.70 (0.51–0.96)	0.03
Dominant model	C/T + C/C	614 (71.6%)	398 (65.7%)	0.76 (0.61–0.95)	0.02	0.75 (0.60–0.94)	0.01
Recessive model	T/T + C/T	697 (81.2%)	508 (83.8%)	Reference		Reference	
	C/C	161 (18.8%)	98 (16.2%)	0.84 (0.63–1.10)	0.20	0.83 (0.63–1.10)	0.19
Overdominant model	T/T + C/C	405 (47.2%)	306 (50.5%)	Reference		Reference	
	C/T	453 (52.8%)	300 (49.5%)	0.88 (0.71–1.08)	0.21	0.87 (0.71–1.08)	0.20
Log-additive model	T	941 (54.8%)	716 (59.1%)	Reference		Reference	
	C	775 (45.2%)	496 (40.9%)	0.83 (0.72–0.97)	0.02	0.83 (0.71–0.97)	0.01

rs1562430							

Codominant model	A/A	594 (67.6%)	415 (68.1%)	Reference		Reference	
	G/A	254 (28.9%)	165 (27.1%)	0.93 (0.74–1.17)	0.54	0.95 (0.75–1.20)	0.65
	G/G	31 (3.5%)	29 (4.8%)	1.34 (0.79–2.26)	0.27	1.20 (0.71–2.04)	0.50
Dominant model	G/A + G/G	285 (32.4%)	194 (31.9%)	0.97 (0.78–1.22)	0.82	0.98 (0.78–1.22)	0.83
Recessive model	A/A + G/A	848 (96.5%)	580 (95.2%)	Reference		Reference	
	G/G	31 (3.5%)	29 (4.8%)	1.37 (0.82–2.29)	0.24	1.22 (0.72–2.06)	0.46
Overdominant model	A/A + G/G	625 (71.1%)	444 (72.9%)	Reference		Reference	
	G/A	254 (28.9%)	165 (27.1%)	0.91 (0.73–1.15)	0.45	0.94 (0.74–1.18)	0.58
Log-additive model	A	1442 (82.0%)	995 (81.7%)	Reference		Reference	
	G	316 (18.0%)	223 (18.3%)	1.02 (0.85–1.23)	0.82	1.01 (0.84–1.22)	0.93

^*∗*^Adjusted for age, age at first full-term pregnancy, menopausal status, and hormonal therapy status.

OR, odds ratio; CI, confidence interval.

**Table 3:** Association between four nongenic polymorphisms and Luminal-A breast cancer risk.

SNP or model	Genotype or allele	Controls (%) (*n* = 882)	Cases (%) (*n* = 263)	Raw OR (95% CI)	Raw *P*	Adjusted OR (95% CI)^*∗*^	Adjusted *P*
rs13387042							

Codominant model	G/G	704 (82.5%)	202 (77.7%)	Reference		Reference	
	G/A	145 (17%)	52 (20.0%)	1.25 (0.88–1.78)	0.22	1.20 (0.84–1.71)	0.32
	A/A	4 (0.5%)	6 (2.3%)	5.23 (1.46–18.70)	0.01	4.67 (1.29–16.91)	0.02
Dominant model	G/A + A/A	149 (17.5%)	58 (22.3%)	1.36 (0.96–1.91)	0.08	1.29 (0.92–1.83)	0.15
Recessive model	G/G + G/A	849 (99.5%)	254 (97.7%)	Reference		Reference	
	A/A	4 (0.5%)	6 (2.3%)	5.01 (1.40–17.91)	0.01	4.51 (1.25–16.30)	0.02
Overdominant model	G/G + A/A	708 (83.0%)	208 (80.0%)	Reference		Reference	
	G/A	145 (17.0%)	52 (20.0%)	1.22 (0.86–1.74)	0.27	1.17 (0.82–1.67)	0.39
Log-additive model	G	1553 (91.0%)	456 (87.7%)	Reference		Reference	
	A	153 (9.0%)	64 (12.3%)	1.43 (1.05–1.95)	0.03	1.36 (1.00–1.87)	0.05

rs981782							

Codominant model	T/T	360 (41.0%)	108 (41.1%)	Reference		Reference	
	G/T	392 (44.6%)	108 (41.1%)	0.92 (0.68–1.24)	0.58	0.93 (0.68–1.26)	0.62
	G/G	127 (14.4%)	47 (17.8%)	1.23 (0.83–1.84)	0.30	1.24 (0.83–1.85)	0.29
Dominant model	G/T + G/G	519 (59.0%)	155 (58.9%)	1.00 (0.75–1.32)	0.97	1.00 (0.76–1.33)	0.98
Recessive model	T/T + G/T	752 (85.5%)	216 (82.1%)	Reference		Reference	
	G/G	127 (14.4%)	47 (17.9%)	1.29 (0.89–1.86)	0.18	1.29 (0.89–1.87)	0.18
Overdominant model	T/T + G/G	487 (55.4%)	155 (58.9%)	Reference		Reference	
	G/T	392 (44.6%)	108 (41.1%)	0.87 (0.65–1.14)	0.31	0.87 (0.66–1.16)	0.34
Log-additive model	T	1112 (63.3%)	324 (61.6%)	Reference		Reference	
	G	646 (36.7%)	202 (38.4%)	1.07 (0.88–1.30)	0.50	1.07 (0.88–1.31)	0.48

rs4415084							

Codominant model	T/T	244 (28.4%)	87 (33.5%)	Reference		Reference	
	C/T	453 (52.8%)	134 (51.5%)	0.83 (0.61–1.13)	0.24	0.82 (0.60–1.12)	0.22
	C/C	161 (18.8%)	39 (15.0%)	0.68 (0.44–1.04)	0.08	0.67 (0.44–1.03)	0.07
Dominant model	C/T + C/C	614 (71.6%)	173 (66.5%)	0.79 (0.59–1.06)	0.12	0.78 (0.58–1.05)	0.11
Recessive model	T/T + C/T	697 (81.2%)	221 (85.0%)	Reference		Reference	
	C/C	161 (18.8%)	39 (15.0%)	0.76 (0.52–1.12)	0.16	0.76 (0.52–1.11)	0.15
Overdominant model	T/T + C/C	405 (47.2%)	126 (48.5%)	Reference		Reference	
	C/T	453 (52.8%)	134 (51.5%)	0.95 (0.72–1.26)	0.72	0.94 (0.71–1.25)	0.69
Log-additive model	T	941 (54.8%)	308 (59.8%)	Reference		Reference	
	C	775 (45.2%)	212 (40.8%)	0.83 (0.67–1.01)	0.08	0.82 (0.66–1.00)	0.048

rs1562430							

Codominant model	A/A	594 (67.6%)	177 (67.3%)	Reference		Reference	
	G/A	254 (28.9%)	71 (27.0%)	0.94 (0.69–1.28)	0.69	0.96 (0.70–1.32)	0.80
	G/G	31 (3.5%)	15 (5.7%)	1.62 (0.86–3.08)	0.14	1.49 (0.78–2.83)	0.23
Dominant model	G/A + G/G	285 (32.4%)	86 (32.7%)	1.01 (0.75–1.36)	0.93	1.02 (0.76–1.37)	0.88
Recessive model	A/A + G/A	848 (96.5%)	248 (94.3%)	Reference		Reference	
	G/G	31 (3.5%)	15 (5.7%)	1.65 (0.88–3.11)	0.13	1.50 (0.79–2.85)	0.22
Overdominant model	A/A + G/G	625 (71.1%)	192 (73.0%)	Reference		Reference	
	G/A	254 (28.9%)	71 (27.0%)	0.91 (0.67–1.24)	0.55	0.94 (0.69–1.28)	0.68
Log-additive model	A	1442 (82.0%)	425 (80.8%)	Reference		Reference	
	G	316 (18.0%)	101 (19.2%)	1.08 (0.85–1.38)	0.53	1.07 (0.84–1.37)	0.57

^*∗*^Adjusted for age, age at first full-term pregnancy, menopausal status, and hormonal therapy status.

OR, odds ratio; CI, confidence interval.

**Table 4:** Association between four nongenic polymorphisms and ER− & PR− & HER2+ breast cancer risk.

SNP or model	Genotype or allele	Controls (%) (*n* = 882)	Cases (%) (*n* = 117)	Raw OR (95% CI)	Raw *P*	Adjusted OR (95% CI)^*∗*^	Adjusted *P*
rs13387042							

Codominant model	G/G	704 (82.5%)	86 (73.5%)	Reference		Reference	
	G/A	145 (17%)	31 (26.5%)	1.75 (1.12–2.74)	0.01	1.68 (1.07–2.65)	0.03
	A/A	4 (0.5%)	0 (0%)	0.00 (0.00–NA)	0.99	0.00 (0.00–NA)	0.99
Dominant model	G/A + A/A	149 (17.5%)	31 (26.5%)	1.70 (1.09–2.66)	0.02	1.64 (1.04–2.58)	0.04
Recessive model	G/G + G/A	849 (99.5%)	117 (100%)	Reference		Reference	
	A/A	4 (0.5%)	0 (0%)	0.00 (0.00–NA)	0.31	0.00 (0.00–NA)	0.31
Overdominant model	G/G + A/A	708 (83.0%)	86 (73.5%)	Reference		Reference	
	G/A	145 (17.0%)	31 (26.5%)	1.76 (1.12–2.75)	0.02	1.69 (1.07–2.67)	0.03
Log-additive model	G	1553 (91.0%)	203 (86.8%)	Reference		Reference	
	A	153 (9.0%)	31 (13.2%)	1.60 (1.04–2.46)	0.04	1.55 (1.00–2.40)	0.048

rs981782							

Codominant model	T/T	360 (41.0%)	43 (36.8%)	Reference		Reference	
	G/T	392 (44.6%)	55 (47.0%)	1.17 (0.77–1.79)	0.46	1.22 (0.79–1.87)	0.37
	G/G	127 (14.4%)	19 (16.2%)	1.25 (0.70–2.23)	0.44	1.28 (0.71–2.30)	0.41
Dominant model	G/T + G/G	519 (59.0%)	74 (63.2%)	1.19 (0.80–1.78)	0.38	1.23 (0.82–1.85)	0.31
Recessive model	T/T + G/T	752 (85.5%)	98 (83.8%)	Reference		Reference	
	G/G	127 (14.4%)	19 (16.2%)	1.15 (0.68–1.94)	0.61	1.15 (0.67–1.96)	0.61
Overdominant model	T/T + G/G	487 (55.4%)	62 (53.0%)	Reference		Reference	
	G/T	392 (44.6%)	55 (47.0%)	1.10 (0.75–1.62)	0.62	1.14 (0.77–1.68)	0.52
Log-additive model	T	1112 (63.3%)	141 (60.3%)	Reference		Reference	
	G	646 (36.7%)	93 (39.7%)	1.13 (0.86–1.49)	0.38	1.15 (0.87–1.51)	0.33

rs4415084							

Codominant model	T/T	244 (28.4%)	41 (35%)	Reference		Reference	
	C/T	453 (52.8%)	54 (46.1%)	0.71 (0.46–1.10)	0.12	0.70 (0.45–1.08)	0.11
	C/C	161 (18.8%)	22 (18.8%)	0.81 (0.47–1.42)	0.47	0.74 (0.42–1.30)	0.29
Dominant model	C/T + C/C	614 (71.6%)	76 (65%)	0.74 (0.49–1.11)	0.15	0.71 (0.47–1.07)	0.11
Recessive model	T/T + C/T	697 (81.2%)	95 (81.2%)	Reference		Reference	
	C/C	161 (18.8%)	22 (18.8%)	1.00 (0.61–1.64)	0.99	0.92 (0.56–1.52)	0.75
Overdominant model	T/T + C/C	405 (47.2%)	63 (53.9%)	Reference		Reference	
	C/T	453 (52.8%)	54 (46.1%)	0.77 (0.52–1.13)	0.18	0.78 (0.53–1.16)	0.22
Log-additive model	T	941 (54.8%)	136 (58.1%)	Reference		Reference	
	C	775 (45.2%)	98 (41.9%)	0.87 (0.65–1.15)	0.33	0.83 (0.62–1.11)	0.21

rs1562430							

Codominant model	A/A	594 (67.6%)	80 (68.4%)	Reference		Reference	
	G/A	254 (28.9%)	31 (26.5%)	0.91 (0.58–1.41)	0.66	0.94 (0.60–1.47)	0.79
	G/G	31 (3.5%)	6 (5.1%)	1.44 (0.58–3.55)	0.43	1.19 (0.47–3.00)	0.71
Dominant model	G/A + G/G	285 (32.4%)	37 (31.6%)	0.96 (0.64–1.46)	0.86	0.97 (0.64–1.48)	0.90
Recessive model	A/A + G/A	848 (96.5%)	111 (94.9%)	Reference		Reference	
	G/G	31 (3.5%)	6 (5.1%)	1.48 (0.60–3.62)	0.41	1.21 (0.48–3.03)	0.69
Overdominant model	A/A + G/G	625 (71.1%)	86 (73.5%)	Reference		Reference	
	G/A	254 (28.9%)	31 (26.5%)	0.89 (0.57–1.37)	0.59	0.93 (0.60–1.45)	0.75
Log-additive model	A	1442 (82.0%)	191 (81.6%)	Reference		Reference	
	G	316 (18.0%)	43 (18.4%)	1.03 (0.73–1.45)	0.88	1.01 (0.71–1.42)	0.97

^*∗*^Adjusted for age, age at first full-term pregnancy, menopausal status, and hormonal therapy status.

OR, odds ratio; CI, confidence interval.
