# Supplementary material for: Associations of Genetic Variants at Nongenic Susceptibility Loci with Breast Cancer Risk and Heterogeneity by Tumor Subtype in Southern Han Chinese Women
Source: Biomed Res Int. 2016 Feb 28;2016:3065493. doi: 10.1155/2016/3065493 (PMC4789034; doi:10.1155/2016/3065493)
Supplement: Supplementary file 1 — Supplementary materials included two tables showing the results from both the univariate and multivariate logistic regression analyses on the association between four nongenic polymorphisms and Luminal-B/triple negative subtypes. [file 3065493.f1.docx]

| **TABLE S1:** Association between four non-genic polymorphisms and Luminal-B breast cancer risk | | | | | | | |
| --- | --- | --- | --- | --- | --- | --- | --- |
| **SNP or model** | **Genotype**  **or allele** | **Controls (%)**  **(n=882)** | **Cases (%)**  **(n=113)** | **Raw OR**  **(95% CI)** | **Raw *P*** | **Adjusted OR**  **(95% CI)*** | **Adjusted *P*** |
| rs13387042 |  |  |  |  |  |  |  |
| Codominant model | G/G | 704(82.5%) | 88 (77.9%) | Reference |  | Reference |  |
|  | G/A | 145(17%) | 25 (22.1%) | 1.38 (0.85-2.23) | 0.19 | 1.36 (0.84-2.19) | 0.21 |
|  | A/A | 4(0.5%) | 0 (0%) | 0.00 (0.00-NA) | 0.99 | 0.00 (0.00-NA) | 0.99 |
| Dominant model | G/A+A/A | 149(17.5%) | 25 (22.1%) | 1.34 (0.83-2.17) | 0.24 | 1.32 (0.82-2.13) | 0.26 |
| Recessive model | G/G+G/A | 849(99.5%) | 113 (100%) | Reference |  | Reference |  |
|  | A/A | 4(0.5%) | 0 (0%) | 0.00 (0.00-NA) | 0.32 | 0.00 (0.00-NA) | 0.32 |
| Overdominant model | G/G+A/A | 708(83.0%) | 88 (77.9%) | Reference |  | Reference |  |
|  | G/A | 145(17.0%) | 25 (22.1%) | 1.39 (0.86-2.24) | 0.19 | 1.37 (0.84-2.21) | 0.21 |
| Log-additive model | G | 1553(91.0%) | 201(88.9%) | Reference |  | Reference |  |
|  | A | 153(9.0%) | 25(11.1%) | 1.28 (0.81-2.03) | 0.30 | 1.26 (0.79-2.01) | 0.33 |
| rs981782 |  |  |  |  |  |  |  |
| Codominant model | T/T | 360(41.0%) | 46 (40.7%) | Reference |  | Reference |  |
|  | G/T | 392(44.6%) | 52 (46.0%) | 1.04 (0.68-1.58) | 0.86 | 1.05 (0.69-1.60) | 0.84 |
|  | G/G | 127(14.4%) | 15 (13.3%) | 0.92 (0.50-1.71) | 0.80 | 0.93 (0.50-1.72) | 0.81 |
| Dominant model | G/T+G/G | 519(59.0%) | 67 (59.3%) | 1.01 (0.68-1.51) | 0.96 | 1.02 (0.68-1.52) | 0.93 |
| Recessive model | T/T+G/T | 752(85.5%) | 98 (86.7%) | Reference |  | Reference |  |
|  | G/G | 127(14.4%) | 15 (13.3%) | 0.91 (0.51-1.61) | 0.74 | 0.91 (0.51-1.61) | 0.74 |
| Overdominant model | T/T+G/G | 487(55.4%) | 61 (54.0%) | Reference |  | Reference |  |
|  | G/T | 392(44.6%) | 52 (46.0%) | 1.06 (0.71-1.57) | 0.78 | 1.07 (0.72-1.58) | 0.75 |
| Log-additive model | T | 1112(63.3%) | 144(63.7%) | Reference |  | Reference |  |
|  | G | 646(36.7%) | 82(36.3%) | 0.98 (0.74-1.30) | 0.89 | 0.98 (0.74-1.31) | 0.91 |
| rs4415084 |  |  |  |  |  |  |  |
| Codominant model | T/T | 244(28.4%) | 42 (37.2%) | Reference |  | Reference |  |
|  | C/T | 453(52.8%) | 54 (47.8%) | 0.69 (0.45-1.07) | 0.10 | 0.98 (0.74-1.31) | 0.09 |
|  | C/C | 161(18.8%) | 17 (15.0%) | 0.61 (0.34-1.11) | 0.11 | 0.60 (0.33-1.09) | 0.09 |
| Dominant model | C/T+C/C | 614(71.6%) | 71 (62.8%) | 0.67 (0.45-1.01) | 0.06 | 0.66 (0.44-1.00) | 0.05 |
| Recessive model | T/T+C/T | 697(81.2%) | 96 (85.0%) | Reference |  | Reference |  |
|  | C/C | 161(18.8%) | 17 (15.0%) | 0.77 (0.45-1.32) | 0.33 | 0.75 (0.44-1.30) | 0.30 |
| Overdominant model | T/T+C/C | 405(47.2%) | 59 (52.2%) | Reference |  | Reference |  |
|  | C/T | 453(52.8%) | 54 (47.8%) | 0.82 (0.55-1.21) | 0.32 | 0.82 (0.55-1.21) | 0.31 |
| Log-additive model | T | 941(54.8%) | 138(61.1%) | Reference |  | Reference |  |
|  | C | 775(45.2%) | 88(38.9%) | 0.76 (0.57-1.02) | 0.07 | 0.75 (0.56-1.01) | 0.06 |
| rs1562430 |  |  |  |  |  |  |  |
| Codominant model | A/A | 594(67.6%) | 74 (65.5%) | Reference |  | Reference |  |
|  | G/A | 254(28.9%) | 34 (30.1%) | 1.07 (0.70-1.65) | 0.74 | 1.09 (0.71-1.68) | 0.70 |
|  | G/G | 31(3.5%) | 5 (4.4%) | 1.29 (0.49-3.43) | 0.60 | 1.21 (0.45-3.22) | 0.71 |
| Dominant model | G/A+G/G | 285(32.4%) | 39 (34.5%) | 1.10 (0.73-1.66) | 0.66 | 1.10 (0.73-1.67) | 0.65 |
| Recessive model | A/A+G/A | 848(96.5%) | 108 (95.6%) | Reference |  | Reference |  |
|  | G/G | 31(3.5%) | 5 (4.4%) | 1.27 (0.48-3.33) | 0.64 | 1.18 (0.45-3.11) | 0.75 |
| Overdominant model | A/A+G/G | 625(71.1%) | 79 (69.9%) | Reference |  | Reference |  |
|  | G/A | 254(28.9%) | 34 (30.1%) | 1.06 (0.69-1.62) | 0.79 | 1.08 (0.70-1.65) | 0.74 |
| Log-additive model | A | 1442(82.0%) | 182(80.5%) | Reference |  | Reference |  |
|  | G | 316(18.0%) | 44(19.5%) | 1.10 (0.78-1.56) | 0.59 | 1.09 (0.77-1.54) | 0.62 |
| * Adjusted for age, age at first full-term pregnancy, menopausal status, and hormonal therapy status.  Abbreviations: OR, odds ratio; CI, confidence interval. | | | | | | | |
| **TABLE S2:** Association between four non-genic polymorphisms and Triple negative breast cancer risk | | | | | | | |
| **SNP or model** | **Genotype**  **or allele** | **Controls (%)**  **(n=882)** | **Cases (%)**  **(n=116)** | **Raw OR**  **(95% CI)** | **Raw *P*** | **Adjusted OR**  **(95% CI)*** | **Adjusted *P*** |
| rs13387042 |  |  |  |  |  |  |  |
| Codominant model | G/G | 704(82.5%) | 96 (82.8%) | Reference |  | Reference |  |
|  | G/A | 145(17%) | 20 (17.2%) | 1.01 (0.61-1.69) | 0.97 | 0.98 (0.58-1.64) | 0.93 |
|  | A/A | 4(0.5%) | 0 (0%) | 0.00 (0.00-NA) | 0.99 | 0.00 (0.00-NA) | 0.99 |
| Dominant model | G/A+A/A | 149(17.5%) | 20 (17.2%) | 0.98 (0.59-1.64) | 0.95 | 0.95 (0.57-1.60) | 0.85 |
| Recessive model | G/G+G/A | 849(99.5%) | 116 (100%) | Reference |  | Reference |  |
|  | A/A | 4(0.5%) | 0 (0%) | 0.00 (0.00-NA) | 0.31 | 0.00 (0.00-NA) | 0.32 |
| Overdominant model | G/G+A/A | 708(83.0%) | 96 (82.8%) | Reference |  | Reference |  |
|  | G/A | 145(17.0%) | 20 (17.2%) | 1.02 (0.61-1.70) | 0.95 | 0.98 (0.59-1.65) | 0.95 |
| Log-additive model | G | 1553(91.0%) | 212(91.4%) | Reference |  | Reference |  |
|  | A | 153(9.0%) | 20(18.6%) | 0.96 (0.58-1.57) | 0.86 | 0.93 (0.56-1.53) | 0.76 |
| rs981782 |  |  |  |  |  |  |  |
| Codominant model | T/T | 360(41.0%) | 55 (47.4%) | Reference |  | Reference |  |
|  | G/T | 392(44.6%) | 45 (38.8%) | 0.75 (0.49-1.14) | 0.18 | 0.76 (0.50-1.15) | 0.20 |
|  | G/G | 127(14.4%) | 16 (13.8%) | 0.82 (0.46-1.49) | 0.52 | 0.83 (0.46-1.51) | 0.54 |
| Dominant model | G/T+G/G | 519(59.0%) | 61 (52.6%) | 0.77 (0.52-1.13) | 0.19 | 0.77 (0.52-1.15) | 0.20 |
| Recessive model | T/T+G/T | 752(85.5%) | 100 (86.2%) | Reference |  | Reference |  |
|  | G/G | 127(14.4%) | 16 (13.8%) | 0.95 (0.54-1.66) | 0.85 | 0.95 (0.54-1.67) | 0.86 |
| Overdominant model | T/T+G/G | 487(55.4%) | 71 (61.2%) | Reference |  | Reference |  |
|  | G/T | 392(44.6%) | 45 (38.8%) | 0.79 (0.53-1.17) | 0.23 | 0.79 (0.53-1.18) | 0.25 |
| Log-additive model | T | 1112(63.3%) | 155(66.8%) | Reference |  | Reference |  |
|  | G | 646(36.7%) | 77(33.2%) | 0.86 (0.65-1.14) | 0.30 | 0.87 (0.65-1.15) | 0.32 |
| rs4415084 |  |  |  |  |  |  |  |
| Codominant model | T/T | 244(28.4%) | 38 (32.8%) | Reference |  | Reference |  |
|  | C/T | 453(52.8%) | 58 (50.0%) | 0.82 (0.53-1.27) | 0.38 | 0.80 (0.51-1.24) | 0.31 |
|  | C/C | 161(18.8%) | 20 (17.2%) | 0.80 (0.45-1.42) | 0.44 | 0.75 (0.42-1.35) | 0.34 |
| Dominant model | C/T-C/C | 614(71.6%) | 78 (67.2%) | 0.82 (0.54-1.24) | 0.34 | 0.78 (0.52-1.19) | 0.26 |
| Recessive model | T/T-C/T | 697(81.2%) | 96 (82.8%) | Reference |  | Reference |  |
|  | C/C | 161(18.8%) | 20 (17.2%) | 0.90 (0.54-1.50) | 0.69 | 0.87 (0.52-1.46) | 0.60 |
| Overdominant model | T/T-C/C | 405(47.2%) | 58 (50.0%) | Reference |  | Reference |  |
|  | C/T | 453(52.8%) | 58 (50.0%) | 0.89 (0.61-1.32) | 0.57 | 0.88 (0.60-1.31) | 0.54 |
| Log-additive model | T | 941(54.8%) | 134(57.8%) | Reference |  | Reference |  |
|  | C | 775(45.2%) | 98(42.2%) | 0.88 (0.66-1.17) | 0.39 | 0.86 (0.64-1.14) | 0.29 |
| rs1562430 |  |  |  |  |  |  |  |
| Codominant model | A/A | 594(67.6%) | 84 (72.4%) | Reference |  | Reference |  |
|  | G/A | 254(28.9%) | 29 (25.0%) | 0.81 (0.52-1.26) | 0.35 | 0.83 (0.53-1.30) | 0.41 |
|  | G/G | 31(3.5%) | 3 (2.6%) | 0.68 (0.20-2.29) | 0.54 | 0.58 (0.17-1.95) | 0.38 |
| Dominant model | G/A-G/G | 285(32.4%) | 32 (27.6%) | 0.79 (0.52-1.22) | 0.29 | 0.80 (0.52-1.23) | 0.29 |
| Recessive model | A/A-G/A | 848(96.5%) | 113 (97.4%) | Reference |  | Reference |  |
|  | G/G | 31(3.5%) | 3 (2.6%) | 0.73 (0.22-2.41) | 0.59 | 0.61 (0.18-2.04) | 0.39 |
| Overdominant model | A/A-G/G | 625(71.1%) | 87 (75.0%) | Reference |  | Reference |  |
|  | G/A | 254(28.9%) | 29 (25.0%) | 0.82 (0.53-1.28) | 0.38 | 0.85 (0.54-1.32) | 0.46 |
| Log-additive model | A | 1442(82.0%) | 197(84.9%) | Reference |  | Reference |  |
|  | G | 316(18.0%) | 35(15.1%) | 0.81 (0.56-1.19) | 0.27 | 0.80 (0.55-1.17) | 0.24 |
| * Adjusted for age, age at first full-term pregnancy, menopausal status, and hormonal therapy status.  Abbreviations: OR, odds ratio; CI, confidence interval.. | | | | | | | |
